# Simulation-based training improves patient safety climate in acute stroke care (STREAM)

**DOI:** 10.1186/s42466-021-00132-1

**Published:** 2021-07-12

**Authors:** Ferdinand O. Bohmann, Joachim Guenther, Katharina Gruber, Tanja Manser, Helmuth Steinmetz, Waltraud Pfeilschifter, for the STREAM Trial investigators

**Affiliations:** 1 University Hospital Frankfurt, Department of Neurology, Goethe University, Theodor-Stern-Kai 7, Frankfurt am Main, Germany; 2 FHNW School of Applied Psychology, University of Applied Sciences and Arts Northwestern Switzerland (FHNW), Olten, Switzerland; 3 Klinik für Neurologie und Klinische Neurophysiologie, Klinikum Lüneburg, Lüneburg, Germany

**Keywords:** Stroke, Acute stroke care, Patient safety, Patient safety climate, SAQ

## Abstract

**Background:**

Treatment of acute stroke performed by a multiprofessional, interdisciplinary team is highly time dependent. Interface problems are preprogrammed and pitfalls relevant to patient safety are omnipresent. The Safety Attitudes Questionnaire (SAQ) is a validated and widely used instrument to measure patient safety. The objective of this study was to evaluate the influence of Simulation-based Training of the Rapid Evaluation and Management of Acute Stroke (STREAM) on patient safety measured by SAQ in the context of acute stroke care.

**Methods:**

During the STREAM trial at seven university hospitals in Germany from October 2017 to October 2018, an anonymous survey was conducted before and after the STREAM intervention centering around interdisciplinary simulation training. The questionnaire, based on the SAQ, included 33 items (5-point Likert scale, 1 = disagree to 5 = agree) and was addressed at the whole multiprofessional stroke team. Statistical analyses were used to examine psychometric properties as well as descriptive findings.

**Results:**

In total 167 questionnaires were completed representing an overall response rate of 55.2%, including especially physicians (65.2%) and nurses (26.3%). Safety climate was significantly improved (pre-interventional: 3.34 ± .63 vs. post-interventional: 3.56 ± .69, *p* = .028). The same applies for teamwork climate among stroke teams (pre-interventional: 3.76 ± .59 vs. post-interventional: 3.84 ± .57, *p* = .001). The perceived benefit was most relevant among nurses.

**Conclusions:**

The STREAM intervention centering around interdisciplinary simulation training increases perceived patient safety climate assessed by the SAQ in acute stroke therapy. These results have the potential to be a basis for future quality improvement programs.

**Supplementary Information:**

The online version contains supplementary material available at 10.1186/s42466-021-00132-1.

## Introduction

Human errors are especially in focus during time critical operations like acute stroke care. Critically ill patients require an advanced level of patient safety. Since the milestone publication ‘To Err is human’ the link between patient safety and human errors has been widely accepted [1]. The involvement of a multiprofessional, interdisciplinary team challenges the institutional safety culture. From an anthropological perspective, the survey of a culture is a long-term observation of norms, beliefs, values, artifacts, symbols, and rituals. Therefore, the term ‘patient safety climate’ is usually used in medicine. Safety climate is the consensus of shared perceptions regarding patient safety norms and behaviors by the staff in a clinical area. Studies have linked safety climate to clinical and operational outcomes [2–5].

In the sense of ‘you can’t improve what you don’t measure’ [6] the Safety Attitudes Questionnaire (SAQ) has been developed and adopted to various clinical settings and validated in different languages [7]. It is the most widely used instrument for measuring patient safety climate [8]. The initial version of the SAQ has 60 items, including 34 core items with six domains (teamwork climate, safety climate, job satisfaction, perception of management, stress recognition, working conditions). Sexton and colleagues have established the analysis of individual domains [9]. For intensive care units (ICU) the SAQ factors have already proven to be sensitive for changes by a quality improvement program, associated with reductions in medication errors and with shorter lengths of stay [10]. It has been also shown that critical care units with highest scores on SAQ factors had the lowest subsequent blood-stream infection rate [7, 11]. Based on real-life studies targeting safety climate [6, 9, 12, 13], the proposed cut-off for each SAQ factor should be 60 point (on a 100 point scale) respectively 3.4 points on the 5-point Likert scale [7, 14]. However, ongoing efforts in the stroke field to improve quality of care and patient safety have not yet been examined using the SAQ. Especially the SAQ dimension teamwork climate, safety climate and job satisfaction seem to be suitable for this purpose and will be investigated in more detail in this pilot study.

Crew resource management (CRM), coined in 1979 by NASA psychologist John Lauber, is targeting the link between human errors and patient safety. CRM strengthens non-technical skills like communication and teamwork to avoid human errors. Similar to CRM, simulation training has been shown to enhance team operations and has been associated with improved clinical outcomes [8, 10, 14].

The STREAM (Simulation-based Training of Rapid Evaluation and Management of Acute Stroke) trial was directed at high level stroke centers in a multicentric, prospective interventional design to assess the effect of a multicomponent quality improvement program [15]. We hypothesized that the implementation of a stroke team algorithm, applying the principles of CRM and stroke team simulation training would improve patient safety climate measured by the SAQ.

## Methods

### Design and setting

From October 1st 2017 to July 1st 2018, a cross sectional survey was conducted at four stroke centers of tertiary care university hospitals (University Hospital Augsburg, Ludwig Maximilians-University Munich, University Medical Centre Hamburg, University Hospital Cologne) as part of the STREAM trial. The trial was coordinated by the Neurovascular Research Group of University Hospital Frankfurt (Goethe University) and had the approval of the ethics committee of Frankfurt University Hospital (ID 433/16) with secondary approvals from the ethics committees of all participating centers.

Following a pre−/ post-intervention design the survey was accomplished before and after the STREAM intervention to assess safety culture improvements. The STREAM intervention is described elsewhere [15] and consisted mainly of a critical peer-to-peer review of each institutions’ stroke protocol with the aim of creating an adapted standard operating procedure (SOP), the introduction to the concepts of simulation training as well as crew resource management (CRM) and two full-day stroke team trainings at each trial site led by the principal investigator’s dedicated stroke team trainers. The in situ simulation for the entire interdisciplinary multiprofessional stroke team included an intensive interdisciplinary debriefing focusing on CRM principles [16].

### Assessing the safety attitudes questionnaire (SAQ) – German version

The SAQ was first developed by Sexton and colleagues [7]. Zimmermann et al. translated and validated the short version of the SAQ into the German language version [17]. The goal of the SAQ is to assess safety culture by surveying employees. When using questionnaires to examine perceptions at the group level, the common term used is climate. We report results from the teamwork climate, safety climate and job satisfaction domain of the SAQ. The study intervention was not designed to affect the other domains of the SAQ (perception of management, stress recognition, working conditions). Individual analysis of each domain has been established by Sexton and colleagues form the beginning [9]. Analyzed items and dimensions (teamwork climate, safety climate, job satisfaction) are illustrated in Table 1: teamwork climate (6 items), safety climate (7 items) and job satisfaction (5 items). Answers to the SAQ items are given on a 5-point Likert scale (1 = disagree strongly, 2 = disagree slightly, 3 = neutral, 4 = agree slightly, 5 = agree strongly).

**Table 1:**
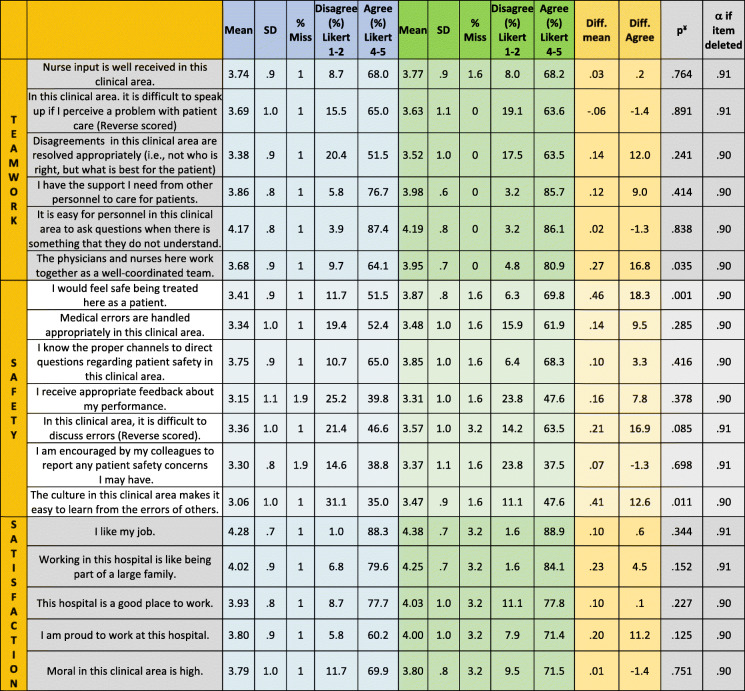
Safety attitudes questionnaire (SAQ) domain item scores

^¥^ Mann-Whitney-U test was used to test statistical significance, nevertheless specific means per question are presented. *Blue background*: pre-intervention; *green background*:: post-intervention. *P* < .05 indicates statistically significance. α (Cronbach’s alpha) if item is deleted is given

### Data collection

In each participating center, all members of the stroke teams (professionals involved in acute stroke care: neurologists, neuroradiologists/ -interventionalists, nurses, medical technical assistants) received an invitation and two e-mail reminders to fill out the German version of SAQ in a paper and pencil version before and after the 6 month period of STREAM intervention. Questionnaires were administered by the local principle investigator, collected and sent back to sponsor (University Hospital Frankfurt) for a central data collection and analysis.

### Statistical analysis

#### Reliability analysis

Factor scale scores were calculated for individual respondents by the taking the average of the specific items per factor. For reliability analysis, Cronbach’s alpha was calculated to assess the internal consistency of factors. Cronbach’s alpha was calculated for each factor (> .7 indicates adequate internal consistency [18]. Separately, scale reliability analysis for each item and dimension resulted in a corrected item-total correlation and Cronbach’s alpha.

#### Descriptive statistics

Frequency tables were used to analyze data and missing values (MV). Scores were reversed for all negatively worded items. Despite the ordinal scaling of SAQ data, the established method is to present results as mean values or percentages (agree/ disagree) [9, 19]. Screening for normal distribution was done with boxplots and q-q plots. To illustrate percentages of participants that agreed or disagreed with each specific item on the 5-point Likert scale, values of 1 and 2 were recoded as ‘disagree’, 3 as ‘neutral’ and 4 and 5 as ‘agree’. A threshold score of 3.4 points on the 5-point Likert scale (representing 60% agreement on the 0 to 100-point scale where disagree strongly becomes 0, disagree slightly becomes 25, neutral becomes 50, agree slightly becomes 75 and agree strongly becomes 100) should be exceeded, with a “goal zone” of 4.2 to 5 points [14].

The primary endpoint of the study was the patient safety climate within the overall multiprofessional, interdisciplinary stroke team. For this purpose, the factors teamwork climate, safety climate and job satisfaction were compared before and after the STREAM study intervention. As secondary endpoints, subgroup analyses with respect to professional position and specialty were added.

For interpretation of group differences, multivariate analyses of variance (MANOVA) were used to analyze mean scores. Separate MANOVA’s (Wilks Lambda) were performed with pre-and post-intervention, professional position and specialty as independent variables. Post-hoc univariate ANOVAs were conducted for every dependent variable. Additionally Tukey HSD post-hoc analysis explored differences between groups.

Statistical significance of group differences was tested via two tailed Student’s t-test or Wilcoxon-Mann-Whitney test. Pearson’s Chi-Square-test was used for nominally scaled values. A *p*-value of < .05 was deemed to indicate significance. Data was analyzed with SPSS 26 (IBM; Armonk, BY, USA) and GraphdPad 9 (GraphPad Software, USA).

## Results

### Reliability and descriptive statistics

The internal consistency of the questionnaire was satisfactory with Cronbach’s alpha .91 for the overall questionnaire. Cronbach’s alpha for the respective individual factor, indicating homogeneity, was above .70 (teamwork climate: .76, safety climate: .81, job satisfaction: .83). Missing values did not exceed 3.2% per item. We found no statistical significant difference for missing value rates between trial centers, specialties or professions (Table 1).

In total, 167 questionnaires (pre-interventional 103, post-interventional 64) were returned by participants representing an overall response rate of 52.2% (pre-interventional 66.3%, post-interventional 42.0%). Details regarding participants in the pre- and post-interventional phase are presented in Table 2.

**Table 2:** Participant characteristics

	Pre	Post	*p*
	*n* = 103	*n* = 64	
Age (years), mean (SD)	34.7 (9.0)	35.1 (9.3)	.794
Female, n (%)	58 (56.3)	28 (44.4)	.190
Profession, n (%)			
Physician	66 (64.1)	43 (68.3)	.674
Nurse	29 (28.2)	15 (23.8)	
Specialty, n (%)			
Neurology	59 (57.3)	50 (79.4)	.009
Neuroradiology	30 (29.1)	8 (12.7)	
Number of years working, mean (SD)			
As physician/ nurse	9.6 (9.1)	9.4 (9.3)	.886
In acute stroke care	6.7 (7.2)	7.6 (7.4)	.462

Chi-Square test respectively Student’s t-test were used, *p* < .05 indicates a significant difference

### Primary outcome perceived patient safety climate

**Fig. 1:**
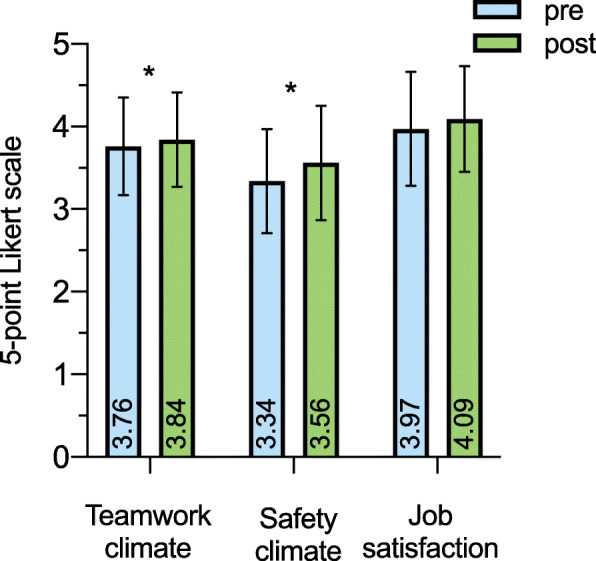
Influence of simulation-based study intervention on patient safety climate. Mean values and standard deviation (SD) are depicted for each SAQ factor before (pre) and after (post) STREAM study intervention. Individual mean values are written vertical per column. The red lines reflect the proposed benchmark of 3.4 points. * significant difference regarding post hoc *analysis p < .05*

Across all domains, the post-interventional SAQ questionnaire score was significantly higher than the pre-interventional score (one-way MANOVA: F(9, 374) = 3.589, *p* < .001, partial η^2^ = .065, Wilk’s Λ = .818), indicating a positive effect of the intervention on overall patient safety climate. Mean scores for teamwork and safety climate were significantly higher in the post-interventional group (Fig. 1, teamwork climate: pre-interventional 3.76 ± .59 vs. post-interventional 3.84 ± .57, *p* = .001, safety climate: 3.34 ± .63 vs. 3.56 ± .69, *p* = .028). For the factor job satisfaction, the small numeric increase was not statistically significant (3.97 ± .69 vs. 4.09 ± .64, *p* = .070). However, the factors teamwork climate and job satisfaction were already scored above the threshold of 3.4 points that is widely-accepted as a surrogate for a positive employee rating before the STREAM intervention. Depending on the profession and specialty, the respective influence of the study intervention on the SAQ factors is shown as subgroup analysis in Table 3.

**Table 3:** Perceptions of patient safety climate per specialty, professional position and working experience

	Teamwork climate		***p***	Safety climate		***p***	Job satisfaction		***p***
	Pre	Post		Pre	Post		Pre	Post	
**Professional position**									
Physician	3.86(.47)	3.86(.56)	.459	3.40(.63)	3.48(.75)	.357	4.01(.60)	4.04(.69)	.564
Nurse	3.51(.70)	3.78(.69)	.114	3.19(.62)	3.78(.54)	.002	3.83(.84)	4.29(.59)	.075
**Specialty**									
Neurology	3.66 (.55)	3.81(.54)	.098	3.20(.59)	3.49(.69)	.008	3.94(.66)	4.03(.67)	.502
Neuroradiology	4.06(.51)	4.33(.33)	.082	3.72(.55)	4.23(.40)	.013	4.17(.68)	4.55(.32)	.130
**In total**	**3.76(.59)**	**3.84(.57)**	**.001**	**3.38(.63)**	**3.56(.69)**	**.028**	**3.97(.69)**	**4.09(.64)**	**.070**

Tukey HSD post-hoc analysis was used, *p* < .05 indicates a significant difference

### Differences in patient safety climate across professions

The influence of the professional group on the respective SAQ factor was examined in a Tukey HSD post-hoc analysis independent of the study intervention respectively distinction in pre- vs. post-intervention. Tukey HSD post-hoc analysis revealed a significant difference for teamwork climate between physicians and nurses (*p* = .018, *M*Diff = .292, 95%-CI[.036, .548]). Safety climate (*p* = .87, *M*Diff = .089, 95%-CI[−.222, .385]) and job satisfaction (*p* = .98, *M*Diff = .030, 95%-CI[−.281, .340]) did not differ significantly between these two professions.

**Fig. 2:**
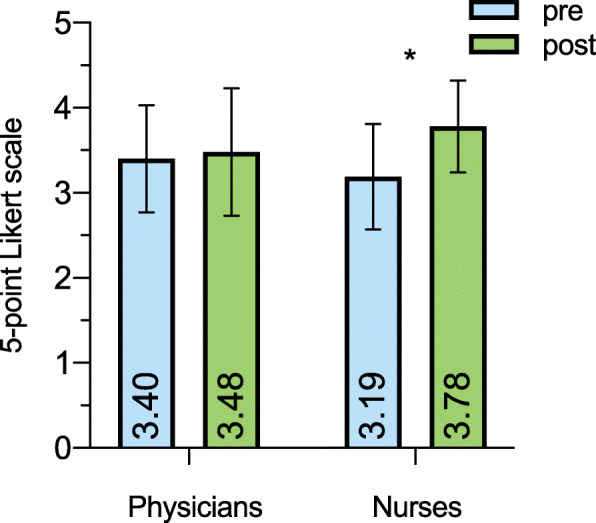
Safety climate depending on profession. Mean values and standard deviation (SD) for perceived safety climate depending on profession are depicted for the pre- and post-interventional phase. Individual mean values are written vertical per column. The red lines reflect the proposed benchmark of 3.4 points. * indicating p < .05

Taking the STREAM study intervention into account, it becomes apparent that especially patient safety perceived by nurses showed a relevant and significant improvement in the post-interventional phase (pre 3.19 ± .62 vs. post 3.78 ± .54, *p* = .002; Fig. 2). For physicians the improvement was weaker (pre 3.40 ± .63 vs. post 3.48 ± .75, *p* = .357).

### Differences in patient safety climate across specialties

In the secondary analysis of the importance of specialty, the factors teamwork climate and patient safety climate were scored significantly higher by neuroradiologists than by neurologists. Without consideration of the study intervention Tukey HSD post-hoc analysis showed a significant higher scores for teamwork climate (*p* = .001, *M*Diff = .376, 95%-CI[.134, .618]) and safety climate (*p* < .001, *M*Diff = .499, 95%-CI[.221, .756]), whereas job satisfaction (*p* = .11, *M*Diff = .250, 95%-CI[−.041, .542]) did not differ significantly.

## Discussion

This study explored the influence of a simulation-based study intervention based on CRM-principles with regard to the perceived patient safety climate in acute stroke care. Our results show that in this pilot trial, significantly higher scores for teamwork climate and patient safety, both cornerstones of a successful team-based stroke care, were reached. In comparison to benchmarking data from emergency departments and intensive care units from other disciplines than neurology, our results indicate comparable results in the field of acute stroke care with the potential for future improvements.

Following the principle ‘you can’t improve what you don’t measure’ [6], our study shows the potential to improve safety climate in acute stroke care. The strength of the SAQ is that the perceived sense of security can be quantified. As has already been shown, this perceived safety is directly linked to patient safety [14]. Based on SAQ validation studies and confirmed by additional real-life studies the proposed cut-off for each SAQ factor should be 60 point (on a 100 point scale) respectively 3.4 points on the 5-point Likert scale used in our study to indicated employees’ confidence adequate patient safety [7, 14]. In the present study, only the factor safety climate did not reach this cut-off prior to the intervention. Following the study intervention, this score was significantly higher and above the benchmark threshold of 3.4 points.

Concerning results for individual SAQ factors, physicians scored higher than nurses in all items except post-interventional safety climate. Interestingly, nurses benefited most from the trial intervention in terms of perceived patient safety (3.19 ± .62 vs. 3.78 ± .54, *p* = .002) resulting in slightly higher scores than physicians in the post-interventional phase. Similar results were found elsewhere [20, 21]. Why nurses are more sensitive to perceive deficits in patient safety has not yet been further evaluated. Greater concerns about patient safety by nurses should be taken into account when designing team trainings in a multiprofessional approach.

Regarding the question whether differences are relevant to attitudes and practice, Pronovost and colleagues demand that improvement measures with regard to safety climate should aim to improve the score by .40 points [14]. This goal was achieved for safety climate among nurses, but not among physicians of different specialties. This could indicate that the study intervention with CRM-based simulation trainings, generally giving each employee only one chance to participate in the training, was underpowered. However, this is contradicted by the fact that the dimension of safety climate as a predefined study objective changed significantly, while job satisfaction were less strongly influenced. This restriction of a pilot study should be considered when planning future studies with a ‘higher dose’ of training.

We acknowledge that there are some limitations of the study results: First, we recruited only four experienced high volume stroke centers for the pre- and post-interventional poll. These centers do probably not reflect the educational needs of stroke teams at primary stroke centers. At the same time, however, this opens the possibility that the effect could be even stronger in less highly educated stroke units with then also greater differences in terms of overall means for SAQ dimension. A disproportionate share of physicians in comparison to nurses is one point further studies should avoid. Therefore, our findings might not be representative for stroke units in general. Second, due to a lower post-interventional response rate, the overall response rate was 52.2% on the SAQ. Therefore, a selection bias cannot be excluded [7]. Third, the study design necessitated a short-term survey after the study intervention. Therefore, we cannot assess long-term effects based on our data. Fourth, due to the single arm design of our trial, it is not possible to infer a direct causal relationship, such as in a cluster-randomized trial. Supplementary qualitative measurements such as structured interviews could help to elicit deeper changes in safety culture.

## Conclusions

A simulation-based study intervention with the focus on CRM principles has the potential to improve teamwork and safety climate of stroke services measured by the German version of the SAQ. In the stroke teams undergoing the survey, nurses had the lowest baseline score of perceived patient safety and the most relevant improvement in the course of the study. Further studies are needed to evaluate the long-term potential of interventional studies for improving patient safety climate in stroke medicine and neurocritical care.

## Supplementary Information

**Additional file 1.** STREAM Collaborators.

## Data Availability

The data that support the findings of this study are available on request from the corresponding author. The data are not publicly available due to privacy or ethical restrictions.
